# Pseudomonas aeruginosa Resists Phage Infection via Eavesdropping on Indole Signaling

**DOI:** 10.1128/spectrum.03911-22

**Published:** 2023-01-05

**Authors:** Guanhua Xuan, Qin Dou, Jiuna Kong, Hong Lin, Jingxue Wang

**Affiliations:** a Food Safety Laboratory, College of Food Science and Engineering, Ocean University of China, Qingdao, China; b College of Marine Life Sciences, Institute of Evolution and Marine Biodiversity, Ocean University of China, Qingdao, China; University of Manitoba

**Keywords:** *Pseudomonas aeruginosa*, phage, indole, type IV pilus, adsorption, quorum sensing

## Abstract

Phage therapy is challenged by the frequent emergence of bacterial resistance to phages. As an interspecies signaling molecule, indole plays important roles in regulating bacterial behaviors. However, it is unclear whether indole is involved in the phage-bacterium interactions. Here, we report that indole modulated phage resistance of Pseudomonas aeruginosa PAO1. Specifically, we found that the type IV pilus (T4P) acts as an important receptor for P. aeruginosa phages vB_Pae_S1 and vB_Pae_TR, and indole could protect P. aeruginosa against phage infection via decreasing the T4P-mediated phage adsorption. Further investigation demonstrated that indole downregulated the expression of genes *pilA*, *pilB*, and *pilQ*, which are essential for T4P assembly and activity. Indole inhibits phage attacks, but our data suggest that indole functions not through interfering with the AHL-based QS pathway, although *las* quorum sensing (QS) of P. aeruginosa PAO1 were reported to promote phage infection. Our finding confirms the important roles of indole in virus-host interactions, which will provide important enlightenment in promoting phage therapy for P. aeruginosa infections.

**IMPORTANCE** Our finding is significant with respect to the study of the interactions between phage and host. Although the important roles of indole in bacterial physiology have been revealed, no direct examples of indole participating in phage-host interactions were reported. This study reports that indole could modulate the phage resistance of indole-nonproducing Pseudomonas aeruginosa PAO1 through inhibition of phage adsorption mechanism. Our finding will be significant for guiding phage therapy and fill some gaps in the field of phage-host interactions.

## INTRODUCTION

Phage therapy is being reevaluated as an alternative to antibiotics for treatment of multidrug-resistant (MDR) bacteria (1). The advantages of phage therapy have been well explained and evaluated elsewhere (2, 3). However, one of the significant obstacles for phage therapy is the evolution of diverse bacterial defense mechanisms under the selective pressure of phage predation, including the CRISPR-Cas system, abortive infection systems, restriction-modification (R-M) systems, and surface modification (4–7).

Quorum sensing (QS) signaling molecules are also involved in regulating gene expression that helps bacteria to resist phage infection. An autoinducer-2 (AI-2)-mediated QS system could inhibit T4 phage infection in Escherichia coli (8). In Vibrio cholera, autoinducer-2 (AI-2) and (S)-3-hydroxytridecan-4-one (CAI-1) can protect *V. cholera* against predatory phages through downregulating phage receptors and promoting the production of hemagglutinin protease (9, 10). Vibrio anguillarum also exhibits downregulation of phage receptor OmpK expression in response to *N*-acyl-l-homoserine lactones (AHL) (11). Indole is considered by some authors to be an intercellular signaling molecule, like a QS signal (12). However, until now, no clear evidence exists regarding the involvement of indole on phage infection.

As a by-product of tryptophan degradation catalyzed by tryptophanase (TnaA), indole is produced by a large number of Gram-positive and Gram-negative bacteria, with its concentrations in human gut of up to 1 mmol/L (13). Indole displays a diverse range of effects on bacterial physiology and metabolism, including the inhibition on QS-controlled bacterial behaviors, such as pigment production, biofilm formation, and virulence (12, 14, 15). Since indole acts as a multifunctional molecule among bacteria, we speculate that the interference of indole on bacterial physiology could also indirectly affect phage infection.

P. aeruginosa is one of the most common causes of healthcare-associated diseases and it displays high resistance to almost all antimicrobial drugs available on the market. Infection with P. aeruginosa leads to diseases with a high mortality rate in patients (16, 17). To orchestrate synchronous production of virulence factors, P. aeruginosa relies on two major QS systems, the *las* and *rhl* systems, which recognize homoserine lactones (AHL) signals (18). P. aeruginosa can't produce indole but its behaviors are regulated by indole (19, 20). In this study, we report that the indole signaling molecule protected P. aeruginosa against phage infection and blocking the phage receptor T4P-mediated adsorption was one of the important mechanisms. Our finding discloses a novel indole signaling molecule-mediated phage resistance mechanism in P. aeruginosa PAO1.

## RESULTS

### Isolation and characterization of P. aeruginosa phages.

**FIG 1:**
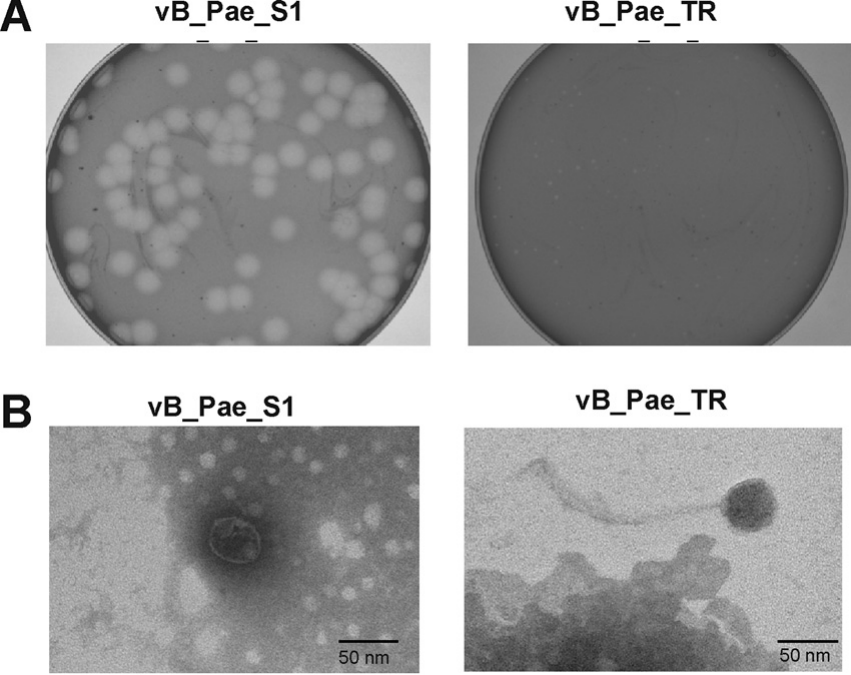
Morphology of phages vB_Pae_S1 and vB_Pae_TR. (A) Plaque morphology of phages vB_Pae_S1 and vB_Pae_TR. (B) Transmission electron microscopy of phages vB_Pae_S1 and vB_Pae_TR. Scale bars: 50 nm.

**FIG 2:**
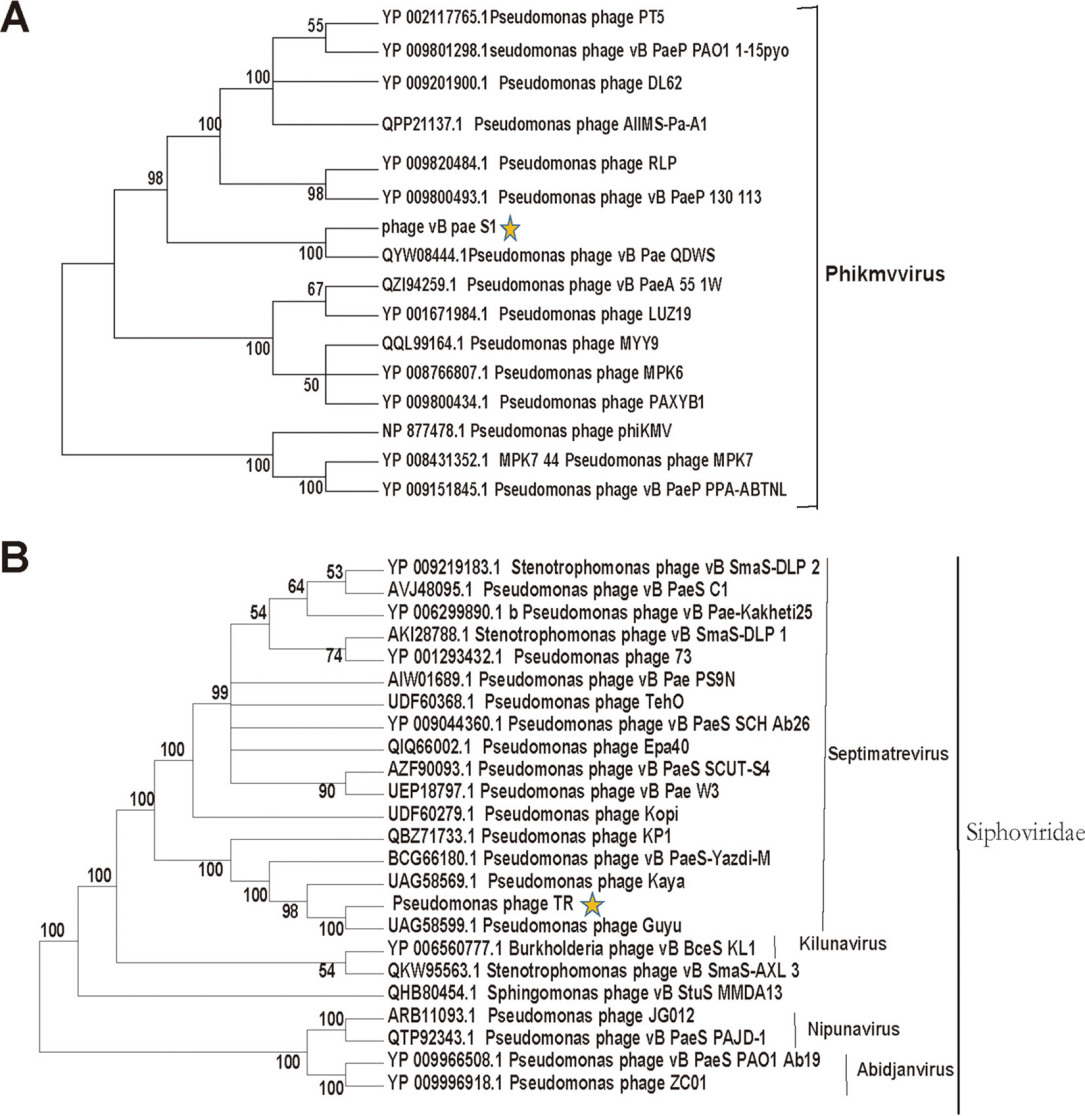
Phylogenetic analysis based on tail fiber protein amino acid sequences of phage (A) vB_Pae_S1 (ORF47) and (B) vB_Pae_TR (ORF05) with related phages. Phylogenetic tree was constructed using the neighbor-joining method using MEGA 7.0 software.

Phage vB_Pae_S1 formed round clear plaques ranged between 5 and 6 mm in diameter on agar plates and vB_Pae_TR produced small plaques (Fig. 1A). Electron microscopy analysis showed that vB_Pae_S1 had a head (51 ± 2 nm) with a stubby tail, classified to the *Podoviridae* family. Phage vB_Pae_TR had a head of approximately 50 nm with a long noncontractile tail of approximately 170 nm, classified to belong to the *Siphoviridae* family (Fig. 1B). Phylogenetic analysis based on the amino acid sequences of tail fiber proteins revealed that vB_Pae_S1 is a member of the genus *Phikmvvirus*, *Krylovirinae* subfamily. Phage vB_Pae_W3 is most closely related to *Septimatrevirus*, *Siphoviridae* (Fig. 2).

### Indole inhibits the synthesis of type IV pilus.

**FIG 3:**
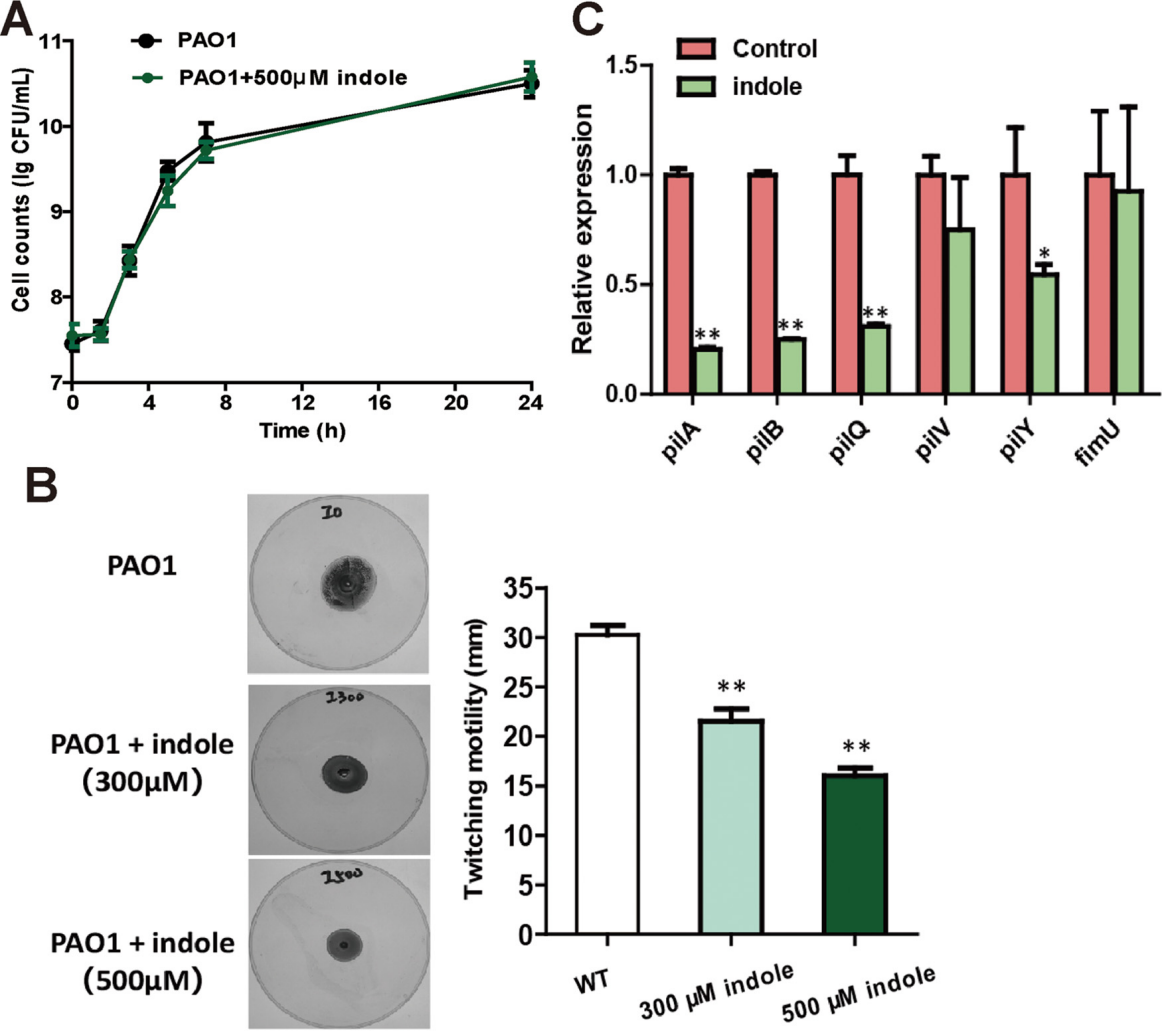
Effects of indole on T4P function of P. aeruginosa PAO1. (A) P. aeruginosa PAO1 growth detection after 24 h cultivation in the presence of 0.5 mM indole or absence of indole. (B) Twitching assay by P. aeruginosa PAO1 with indole being added. Images representative of three independent experiments are shown. (C) RT-qPCR was used to quantify the expression of T4P-related genes in PAO1 and indole-treated (0.5 mM) PAO1. Strains were sampled at an OD_600_ of 1 for RNA extraction. The *rplS* transcript was used as a reference to calculate the relative expression. Data are averages of three experiments with standard deviations. A *t* test was performed (**, *P* < 0.01).

We determined whether exogenous indole had any toxic effect on bacterial growth. Our results show that indole alone did not lead to any adverse effect on cell growth (Fig. 3A). P. aeruginosa PAO1 exhibited significantly decreased twitching motility when treated with the signaling molecule indole (Fig. 3B). Since twitching motility is closely associated with type IV pilus (T4P), mutations in key genes for T4P biosynthesis would make P. aeruginosa lose the twitching motility function (21, 22). PilA, PilB, PilQ, PilV, PilY, and FimU are reported to be critical for T4P assembly (23, 24). RT-qPCR results indicated that the transcripts of *pilA*, *pilB*, and *pilQ* were significantly decreased when 500 μM indole was added. The expression level of *pilY* was also lower in indole-treated group than that in P. aeruginosa PAO1. No changes in *pilV* and *fimU* expression level were found (Fig. 3C). Thus, indole regulated twitching motility of P. aeruginosa via inhibiting the synthesis of T4P.

### Indole signals inhibit phage infection by impairing adsorption.

**FIG 4:**
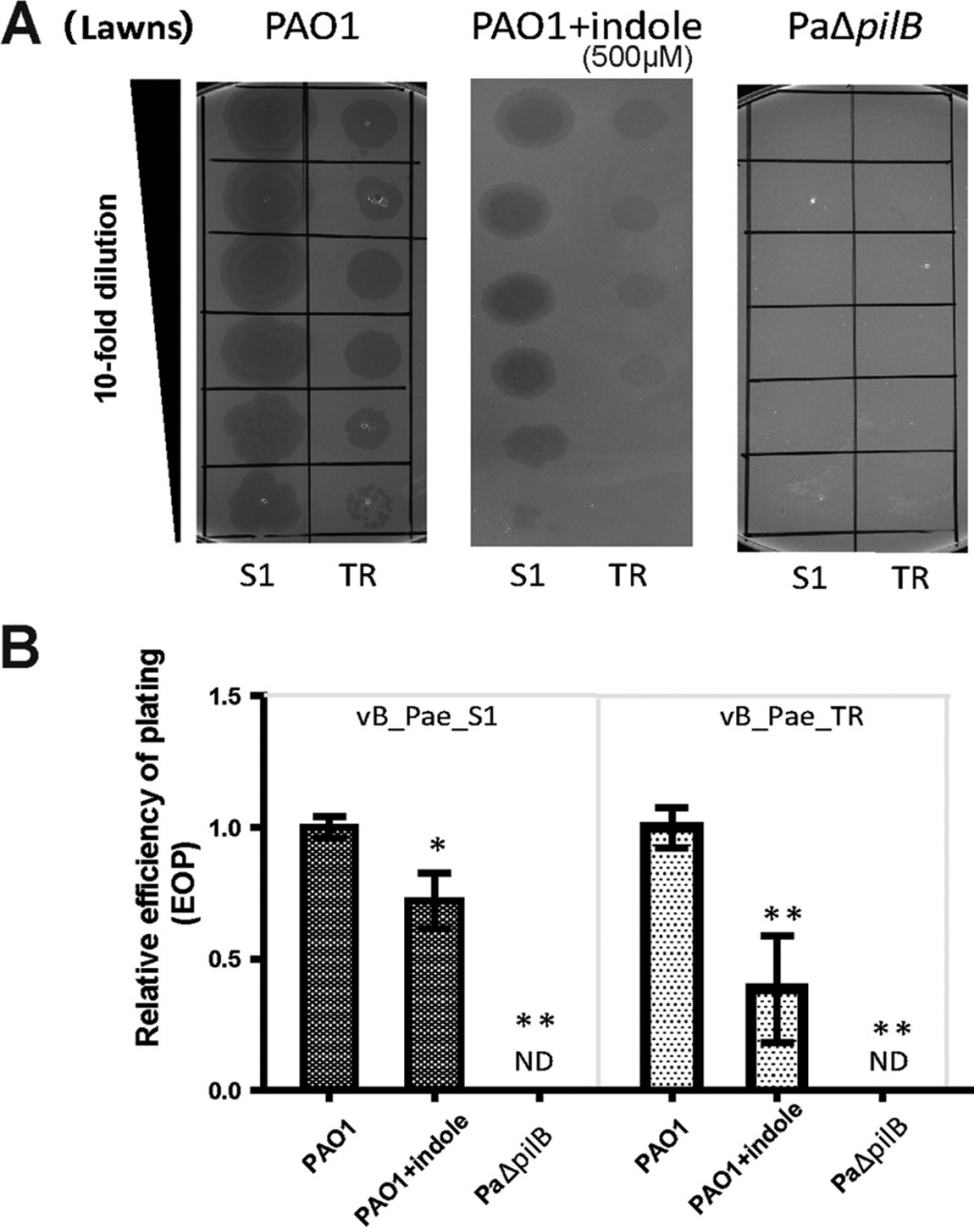
Effects of indole on phage infection were determined by spot assay. (A) Images of plaque formation using diluted effluents on lawns of different P. aeruginosa strains. (B) Relative efficiency of plating (EOP) of phages vB_Pae_S1 and vB_Pae_TR on P. aeruginosa strains. The values were the averages of three measures with standard deviation. One asterisk (*) indicates the sample is different (0.01 < *P < *0.05) from the control PAO1, and two asterisks (**) indicate the sample is significantly different (*P < *0.01) from PAO1 (Student’s paired *t* test).

**FIG 5:**
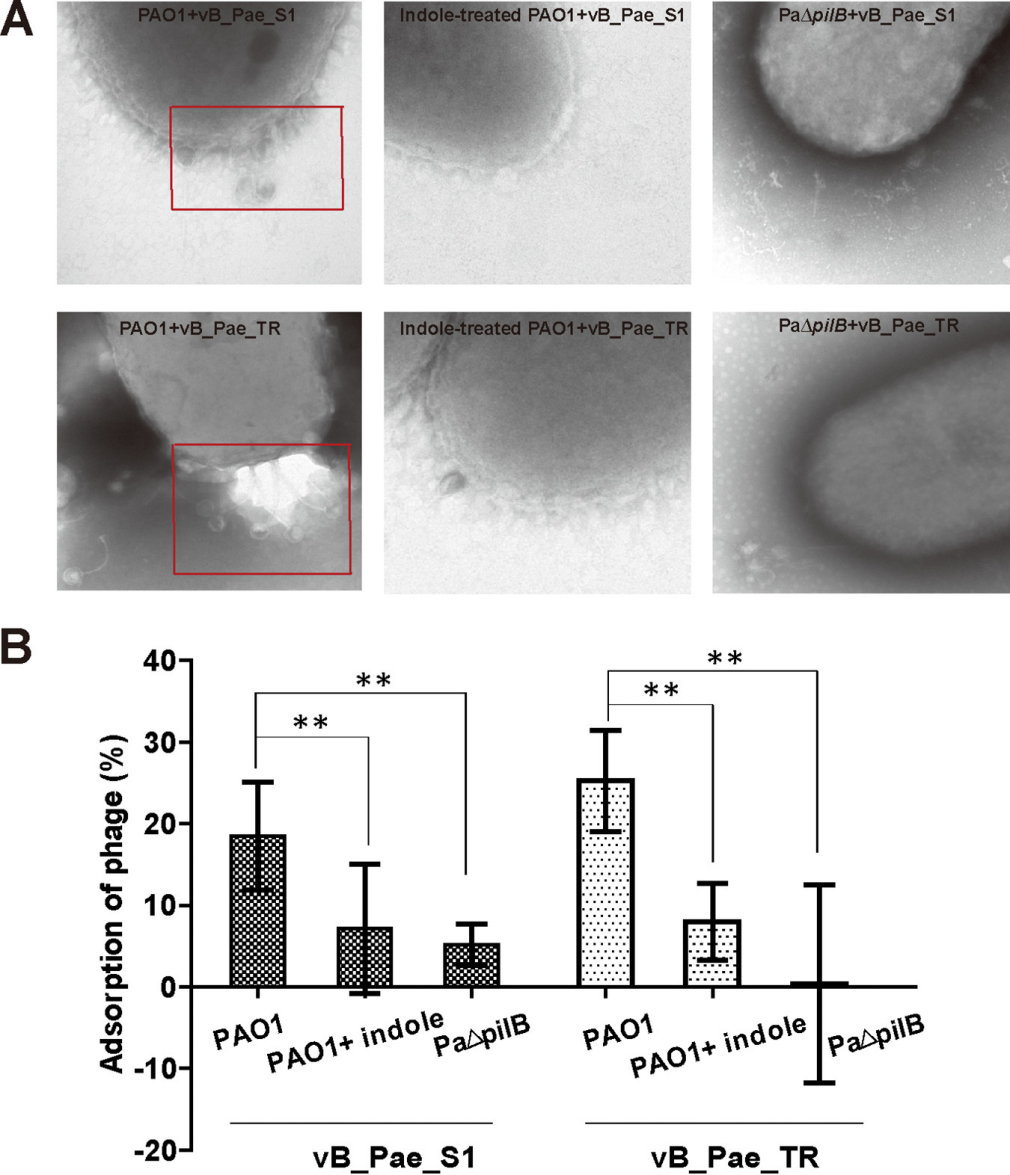
Adsorption of phage vB_Pae_S1 and vB_Pae_TR to P. aeruginosa strains was inhibited by indole. Data are averages of three experiments with standard deviations. A *t* test was performed (**, *P* < 0.01).

Phages vB_Pae_S1 and vB_Pae_TR showed a lower efficiency of plating on the lawns of P. aeruginosa PAO1 supplemented with indole compared to the number of plaques formed on P. aeruginosa PAO1 (Fig. 4A and B). When measuring the cell counts after exposure to phages vB_Pae_S1 and vB_Pae_TR, we found the indole-treated PAO1 are more resistant to phages (data available on request), which all suggested that indole could protect PAO1 against phage infection. By constructing T4P-defective mutants PaΔ*pilB* and performing a spot test, we found phage vB_Pae_S1 and vB_Pae_TR can't infect PaΔ*pilB*, indicating T4P is essential for phage infection. T4P is located on the cell surface and acts a common receptor for many P. aeruginosa phages (25, 26). We speculate that indole functions by blocking T4P-mediated phage adsorption. Results of TEM analysis showed that fewer phages adsorbed on the surface of PaΔ*pilB* and indole-treated PAO1 strains compared with PAO1 (Fig. 5A). By adsorption assay, we also found the adsorption was impaired for PaΔ*pilB* and indole-treated PAO1 following incubation with the phage vB_Pae_S1 and vB_Pae_TR (Fig. 5B). Thus, indole signals could increase phage resistance of P. aeruginosa via decreasing T4P-mediated phage adsorption.

### Indole does not function through interfering with AHL-based QS.

**FIG 6:**
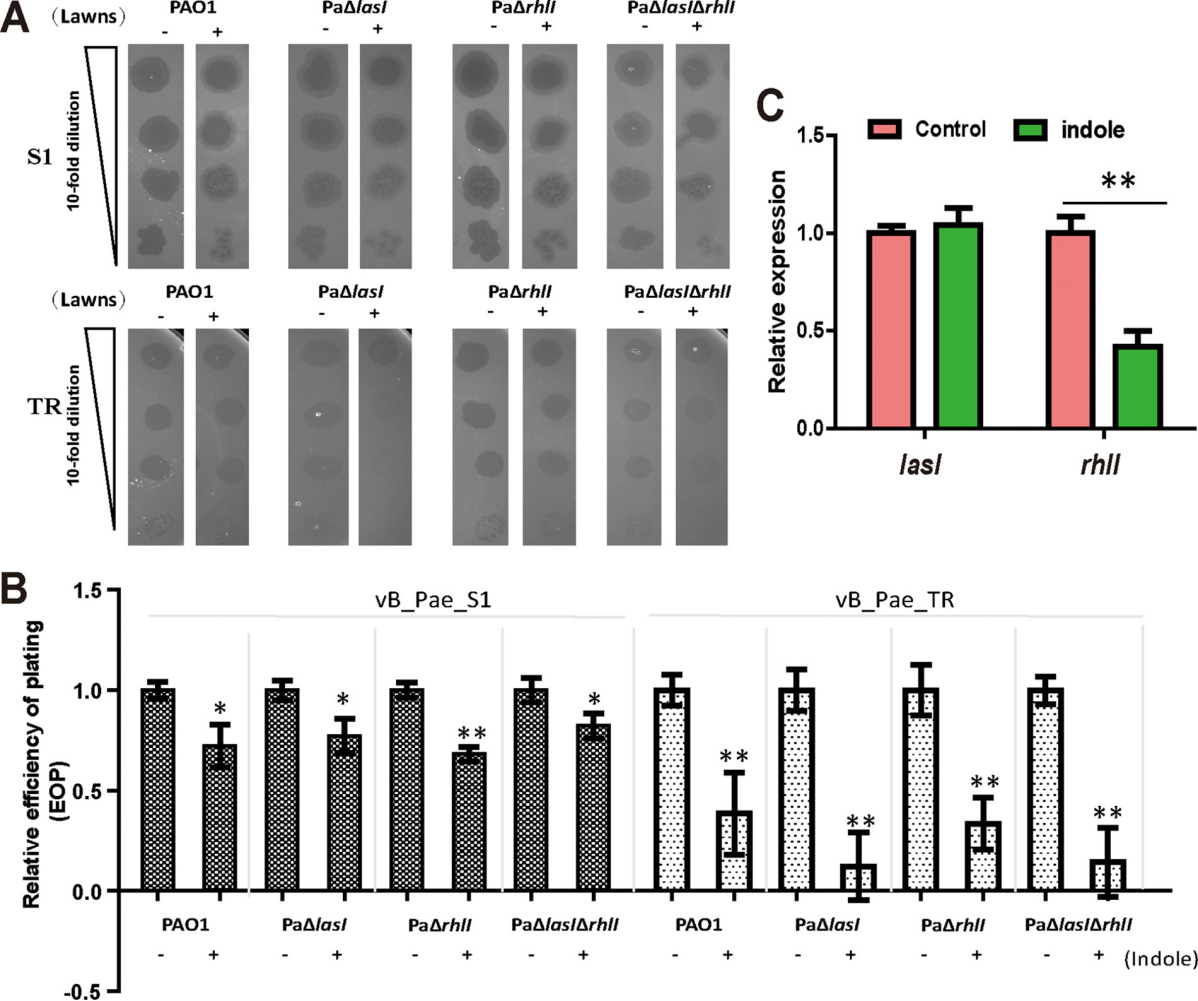
The inhibition of indole on phage infection was not through AHL-based QS. (A) Spot test to determine the effect of indole on phage resistance of different P. aeruginosa mutant strains. (B) Relative EOP of phages vB_Pae_S1 and vB_Pae_TR on P. aeruginosa strains. The values were the averages of three measures with standard deviation. One asterisk (*) indicates the indole-treated strain is different (0.01 < *P < *0.05) from the untreated P. aeruginosa strain, and two asterisks (**) indicate the indole-treated strain is significantly different (*P < *0.01) from the untreated P. aeruginosa strain (Student’s paired *t* test). (C) RT-qPCR was used to quantify the expression of *lasI* and *rhlI* in PAO1 and indole-treated PAO1 when cells grew to an OD_600_ of 1. Data are averages of three experiments with standard deviations. A *t* test was performed (**, *P < *0.01).

To examine whether indole functions via interfering with QS or not, we investigated the inhibition effects of indole on phage infection based on the AHL-based QS mutants. Deletion of *lasI* increased the resistance of P. aeruginosa PAO1 to phage infection, as the transparency of plaques obviously decreased (Fig. 6A). In the CFU reduction assay, we found that the cell counts of both PAO1 and PaΔ*lasI* decreased after a 60-min infection with phage vB_Pae_S1, but then PaΔ*lasI* grew faster than PAO1 (data available on request). When infected with phage vB_Pae_TR, bacterial counts of PaΔ*lasI* reduced by 1.12 log_10_ CFU/mL, while the control PAO1 was decreased by 2.05 log_10_ CFU/mL after a 360-min infection (data available on request). Thus, our data suggest that *las* QS could promote phage infection. However, deletion of *rhlI* had no effect on phage infection. When exogenous indole was added, no loss of the inhibiting effects of indole occurred in PaΔ*lasI*, PaΔ*rhlI*, or PaΔ*lasI*Δ*rhlI* (Fig. 6A). Results of the efficiency of plating (EOP) assay also suggested a reduction in EOP of the two phages vB_Pae_S1 and vB_Pae_TR on indole-treated strains compared with that on the untreated P. aeruginosa group (Fig. 6B). Further inspection of indole on the expression level of *lasI* and *rhlI*, results showed that the *rhlI* expression was decreased, while there was no change in *lasI* expression (Fig. 6C). These results suggest that indole signals do not inhibit phage infection through interfering with AHL-based QS.

## DISCUSSION

**FIG 7:**
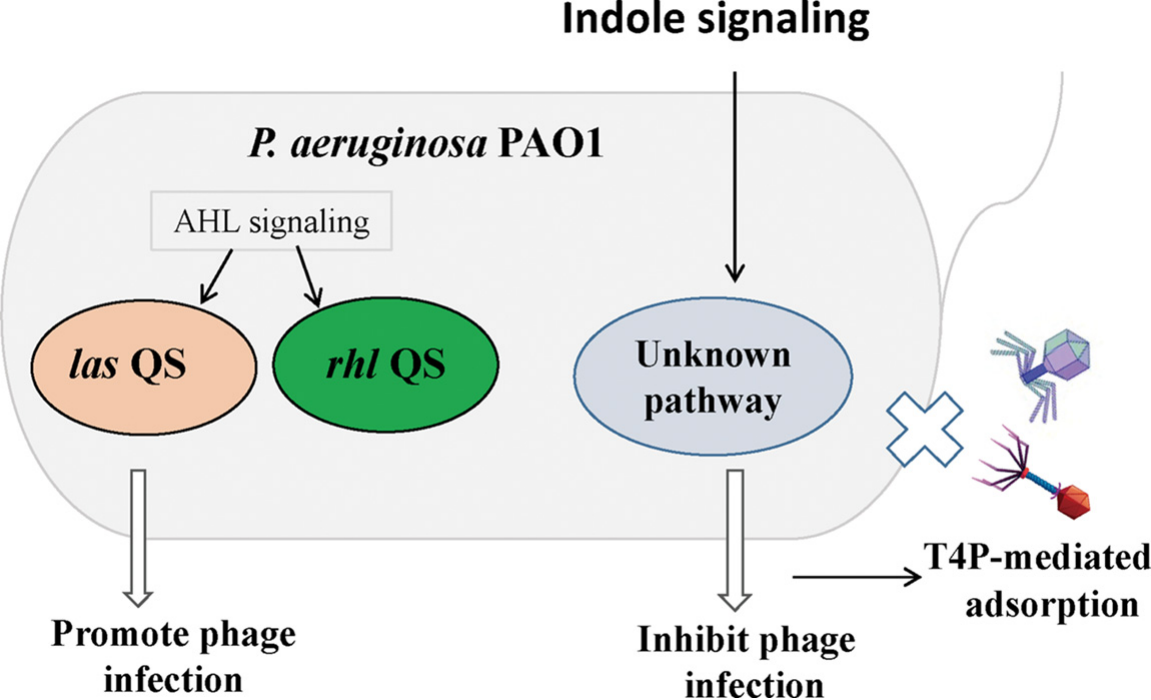
Schematic representation of indole promoting phage resistance in P. aeruginosa PAO1.

Indole controls various physiological functions in indole-producing bacteria, indole-nonproducing bacteria, or in both (14). Here, we demonstrate that indole also performs important roles in phage infection. Phage adsorption is an essential step for phage infection (27). We verified that indole could inhibit phage adsorption via reducingT4P gene expression or probable assembly of functional pili (Fig. 3 and 5). The disruption of *las* QS inhibited phage infection; however, the phage resistance was further increased with added indole (Fig. 6), implying that indole was utilized by P. aeruginosa PAO1 to defend against phage infection through unknown pathways rather than by interfering with AHL-mediated QS pathways (Fig. 7).

PilA, PilB, and PilQ are reported to be essential for T4P assembly and activity, and closely related to T4P-mediated phage infection. Recent work suggest that the glycosylation of PilA could mask potential phage binding surface and block phage infection (28). PilB acts as a signaling protein that could regulate phage infection in response to c-di-GMP signals (29). PilQ is a member of the secretin family of outer membrane proteins; its mutation could also block phage infection (30). Our data show that the expression level of PilA, PilB, and PilQ were significantly decreased when treated with indole (Fig. 3C). And phage adsorption rates were reduced in the indole-treated group compared to PAO1 (Fig. 5). Since T4P acts as an important cell surface receptor for many P. aeruginosa phages (25, 26, 31, 32), our data suggest that inhibiting the T4P-mediated phage adsorption is likely a common mechanism for indole that promotes phage resistance.

Indole globally affects bacterial physiology, including modulating QS. Indole downregulates QS-related virulence factors of P. aeruginosa by altering gene expression (20, 33). Indole is also reported to affect the stabilization of QS regulators, such as TraR (34), that require an AHL signal for stabilization. However, indole regulating bacterial resistance to phage is probably not through QS pathway (Fig. 6). *Las* QS of P. aeruginosa PAO1 promotes phage infection (35), which is consistent with our observation that deletion of *lasI* increased the resistance of P. aeruginosa PAO1 to phage infection (Fig. 6A). However, there was no change in *lasI* expression when treated with indole, but a reduction in *rhlI* expression was found. There was no correlation between *rhl* QS and phage infection (Fig. 6C). Thus, our data suggest that indole regulated phage infection via an unknown pathway within bacterial cells.

It has been reported that the protein receptor was an important target of indole response. Indole could decrease E. coli biofilms via the QS regulator SdiA (36). However, subsequent studies have corrected this, suggesting that SdiA does not respond to indole, though indole can inhibit SdiA activity (37). Indole favors a lipid environment. The envelope stress response modules, BaeSR and CpxAR, were also reported to respond to indole, which supports that the cell membrane might be an important target that indole response (38–40). However, there is no direct example that can support this hypothesis. Although indole has diverse effects on key processes, there is no clearly defined target or mode of action for indole in the bacterial cell (41). Our discovery that indole regulates phage infection in the QS-independent pathway might have some implications for the identification of receptors and pathways for indole signaling.

Phages are tremendously diverse and are found in a diverse range of habitats (42). In response to phage invasion, P. aeruginosa could fully employ QS to control expression of the CRISPR-Cas immune system (43, 44), which is found in 48% of eubacteria and 95% of archaea (45), to protect from phage infection. However, this QS-mediated antiphage pathway is not always efficient, especially for those P. aeruginosa strains that do not possess the CRISPR-Cas system (35). In addition, P. aeruginosa QS systems could activate the expression of the CRISPR-Cas immune system, but also result in increased expression of the phage receptor, such as T4P (46) and lipopolysaccharide (35), which serve as receptors for most Pseudomonas phages characterized to date (32). To reduce the cost of phage resistance and improve the efficiency of antiphage defense, it is unsurprising for the host to resist phage attacks via eavesdropping on exogenous indole signaling molecules.

In summary, our study established the close relationship between indole signaling and phage infection. P. aeruginosa does not produce indole, but its physiological behaviors are regulated by indole (19). Our results indicate that P. aeruginosa PAO1 can “eavesdrop” on environmental indole signaling that protects bacteria against phage infection, but the detailed regulatory pathway of indole on phage resistance warrants further investigation.

## MATERIALS AND METHODS

### Strains, plasmids, and growth conditions.

Standard P. aeruginosa PAO1 strains were cultured in Luria-Bertani (LB) medium at 37°C, with shaking at 200 rpm, unless otherwise stated. The detailed information of strains and plasmids used in this work as well as all primers are available upon request.

### Isolation and purification of phages.

Two phages, vB_Pae_S1 and vB_Pae_TR, were isolated from sewage samples in Qingdao, China, using P. aeruginosa PAO1 as the host by double-layer agar plating method. Single plaques were separated and resuspended in sterile SM buffer (Sterile Solution) (100 mM NaCl, 8 mM MgSO_4_, 50 mM Tris-HCl, pH 7.5). Then, the purified phage was propagated by mixing 1% of overnight culture of PAO1 in liquid LB, then cultured at 37°C for 8 h. Finally, the culture was centrifuged, and the supernatant was filtered to obtain purified phages.

### Transmission electron microscope analysis.

Phages were observed by transmission electron microscope (TEM) as described previously (47). Briefly, high-titer phage suspension (10^11^ PFU/mL) were loaded onto a carbon-coated copper grid for 5 min and negatively stained with 2% phosphotungstic acid (pH 6.8). After drying, the samples were observed using a JEM-1200 EX transmission electron microscope (JEOL) at 100 kV. To observe the phage adsorption by host, P. aeruginosa cells were harvested until grown to an OD_600_ of 2.5, and the cells were then mixed with phages vB_Pae_S1 and vB_Pae_TR, with a multiplicity of infection (MOI) of 100 and 10, respectively. After 5 min of adsorption, the samples were subjected for TEM analysis.

### Gene sequencing and bioinformatic analysis.

Genomic DNA of phages were extracted using the Gene JET genomic DNA purification kit (Thermo Fisher). Genome sequencing was performed by the BGI MGISEQ-2000 platform (BGI Shenzhen, China) to obtain paired-end reads. The filtered reads were assembled into the final genomes with SPAdes v3.13.0.24. The genes and gene products were predicted using the RAST server (http://rast.nmpdr.org/rast.cgi) and verified using NCBI ORF Finder (https://www.ncbi.nlm.nih.gov/orffinder/). Putative protein functions were analyzed and annotated by searching against the nonredundant protein database with BLASTp (http://blast.ncbi.nlm.nih.gov/). Phylogenetic analysis based on the tail fiber proteins of phage vB_Pae_S1 and vB_Pae_TR was constructed by MEGA 7.0 using neighbor joining with a pairwise deletion, p-distance distribution, and bootstrap analysis of 1,000 repeats as the parameters.

### Generation of gene deletion.

All deletions in P. aeruginosa PAO1 were performed according to a published method (48). Briefly, the upstream and downstream fragments of each gene were cloned into pK18mobsacB_tet_ vector to generate pK18mobsacB_tet_-ΔG. The resulting plasmids were transferred into PAO1 via conjugation. Confirmed colonies were subjected to a second round of crossover to produce the gene deletion mutants.

### Phage resistance assay.

Phage resistance was evaluated by spot assay. A total of 3 μL of each phage dilution were spotted on the P. aeruginosa lawn. Indole was stored in dimethyl sulfoxide (DMSO) and added in the melted 1% agar LB medium to form the double-layer agar to a final concentration of 500 μM. An equivalent volume of DMSO was added as a solvent control. The plates were incubated at 37°C for 10 h before examination with a BIO RAD Universal Hood II gel imaging system (Bio-Rad Laboratories, Inc., CA, USA).

The efficiency of plating (EOP) assay was performed as follows. First, 100 μL P. aeruginosa cultures (collected at an OD_600_ = 1) were added in the melted 1% agar LB medium to form the double-layer agar. Indole (500 μM) were added as required. Then, 3 mL of the phage suspension (vB_Pae_S1 and vB_Pae_TR) with serial dilutions were spotted into the lawn of each P. aeruginosa strain. The plates were incubated for 10 h at 37°C, and the number of PFU was counted. Finally, the relative EOP was calculated (average PFU on target bacteria/average PFU on the control P. aeruginosa strain).

### Twitching-motility detection.

Twitching motility assays were modified from a previously described study (47, 49). Briefly, P. aeruginosa strains were stab inoculated to the bottom of an LB 1% agar plate with a P10 pipette tip, and plates were incubated upside down at 37°C for 40 h. Then, agar was carefully removed, and the plastic petri dish was stained with 1% crystal violet for 20 min. Excess dye was washed away with water, and twitching zone diameters were quantified.

### Adsorption rate assay.

P. aeruginosa strains were inoculated in fresh LB medium to an OD_600_ of 1, following a 10-fold dilution in LB. Then, 0.5 mL of phage solution corresponding to vB_Pae_S1 and vB_Pae_TR were mixed with the host cell (0.5 mL) at an MOI of 0.01 and 0.04, respectively. The mixture was incubated at 37°C for 10 min to allow adsorption. LB broth mixed with phages without the bacteria was used as a control. Cultures were then centrifuged at 10,000 × *g* for 2 min and the titers of free phage in the supernatant were determined using the double-layer agar method.

### Real-time quantitative reverse transcription-PCR.

P. aeruginosa strains were grown in LB medium and harvested until grown to OD_600_ = 1. RNA was isolated using the TRIzol RNA purification kit (12183555, Invitrogen) following the manufacturer’s instructions. Total cDNA was synthesized by the HiScript II reverse transcriptase (Vazyme). RT-qPCR was performed by using the SYBR Green Realtime PCR master mix and the StepOnePlus real-time PCR system (ABI). For calculation the relative expression levels of tested genes, *rplS* was used as the reference gene. All experiments were repeated three times.

### Data availability.

All data within the paper are available from the authors upon request. The complete genome sequence of phages vB_Pae_S1 and vB_Pae_TR are available in GenBank under accession numbers OL802210 and OL802211, respectively.
